# Machine learning-enhanced multi-band metamaterial sensor for early detection of neurological disorders

**DOI:** 10.1038/s41598-026-39127-w

**Published:** 2026-02-06

**Authors:** Asad Miah, Sams Al Zafir, Joyonta Das, Jonayed Al-Faruk, Md. Hasnain, Shadi Ihtiaj Zim, S. M. Anowarul Haque, Abdul Wahed

**Affiliations:** 1 Mymensingh Engineering College, Electrical and Electronic Engineering, Mymensingh, 2200 Bangladesh; 2 Mymensingh Engineering College, Computer Science and Engineering, Mymensingh, 2200 Bangladesh

**Keywords:** Engineering, Materials science, Optics and photonics, Physics

## Abstract

This study presents a square enclosed double octagonal shaped metamaterial absorber for the detection of initial neurological conditions. The size is very compact ($0.54 \lambda _0 \times 0.54 \lambda _0 \times 0.05 \lambda _0$) and achieves an absorption value over 99% for frequencies of 4.632 THz, 6 THz, and 7.384 THz. The key focus of the study is sensitivity, which is excellent for all of the peaks, which are 1.5 THz/RIU, 1.5 THz/RIU, and 1.8 THz/RIU, indicating its superior performance across multiple frequency bands. Furthermore, the q factor values are 22.21, 29.41, and 33.81, and the fom values are 7.19, 7.35, and 8.24 for the three frequency peaks, respectively, supporting its reliability in sensing. The electric field, magnetic field, and surface current are analyzed, and an equivalent circuit is created and evaluated for design validation. Additionally, machine learning techniques were employed to optimize and predict the structure’s performance, with the GradientBoosting model demonstrating a reduction of up to 60% in simulation time while maintaining high predictive accuracy. The overall performance and sensing tests across various brain tissues demonstrate excellent results, indicating that this sensor may be an ideal choice for the detection of early-stage neurological disorders.

## Introduction

Cerebrospinal fluid (CSF) is a transparent fluid formed from plasma that fills the ventricles of the brain as well as the subarachnoid spaces that surround the brain and spinal cord^1^. CSF facilitates communication between the central nervous system (CNS) and other systems such as peripheral nerves, lymphatic channels, blood vessels, and the immune system, as well as providing mechanical protection and ensuring internal stability (homeostasis)^2^. A decrease in water content in CSF increases its refractive index, which may suggest or contribute to a variety of neurological disorders as illustrated in Table 3^3,4^. These illnesses can affect the brain and produce a variety of disorders, including acute injury, infections, tumours, and injuries. These diseased brain tissues can cause necrosis, degeneration, or inflammation, and their size and location vary. As a result, their early and accurate detection is crucial for identifying and treating neurological illnesses, such as tumors, traumatic brain damage, and multiple sclerosis^5,6^. Currently, CT scans and MRIs are the primary standards because they can provide detailed images of the brain anatomy, allowing the size and location of damaged brain tissue to be accurately determined. Additionally, imaging techniques can help to identify its type^7^. However, this approach has several disadvantages, including the need for expensive and heavy equipment, as well as the fact that it may not always provide good sensitivity in detecting the early stages of these pathological tissues and their size^8^. Terahertz waves are ideal for biomedical applications because of their nonionizing nature, deep penetration into biological tissues, excellent spatial resolution, and ability to detect unique spectral signatures (fingerprints) of proteins^9^. Metamaterial sensors can perform better in biological sensing because their high absorption value and sensitivity^10^.

A metamaterial is a synthetic substance that has characteristics not observed in naturally occurring materials. Its qualities are determined by the structure and arrangement of subwavelength elements rather than the composition of the material. It is the focus of research in a number of fascinating fields because of its special qualities, including energy harvesting, biological sensors, absorbers, oil sensors, materials thickness sensing applications, and cloaking devices^11–17^. Because of its exceptional sensitivity performance in the terahertz band, metamaterial sensor research in the biomedical field is growing constantly. For biomedical sensing, an adjustable mtm absorber in the THz region has been suggested^18^. Two firm absorption peaks at 98.32 and 98.49 at 4.3 and 7.35 THz were discovered in this investigation. Their fom and q-factor values are 1.55 and 8.92, respectively, and their highest sensitivity is 1.15 THz/RIU. A biosensor for microorganism detection that operates in the 0-3.8 THz range has been demonstrated^19^. They discovered four absorption peaks, with 98.5 being the lowest and 99.95 being the highest. Additionally, with the sensor use for a holder holder, various pesticide types were tested with their refractive index values and frequency shifting values of 103 and 95 GHz were discovered. A THz metamaterial structure had been suggested for use in the biomedical field^20^. This study’s absorption value at 1.1 THz is 98%. Additionally, the sensing performance was tested, yielding a value of 1.08 GHz/g/dL and a q factor and fwhm values of 13.2 and 140 for changes in the Hb concentration. In the THz Fields, a single-layer quarter-ring pattern mtm sensor was demonstrated in^21^ for a variety of uses, including biological sensing. Four transmittance values were found in this study, ranging from 38.4 to 93.7 at frequencies of 0.48, 0.64, 0.79, and 1.04 THz. Furthermore, the q factor values are 6.76, 9.63, 15.51, and 26.28, respectively, and the sensitivity for various refractive index values was also assessed and determined to be 6.84, 7.42, 8.22, and 32.11 GHz/RIU. Considering that blood cancer is one of the most critical issues pertaining to human health, a metamaterial-based sensor was demonstrated in the THz field^10^. This sensor’s operational range is between 0.6 and 1.2 THz, and five absorption peaks are detected between 85% and 99.7%. Additionally, this study tested the sensor’s performance and discovered values of 0.435, 0.7, and 1.29 THz/RIU. Then, using their sensor and electric and magnetic field analysis, they evaluated the microwave imaging technique to identify healthy and malignant cells. An MTM-based biosensor for the identification of cervical cancer cells in their early stages was proposed in^22^. This investigation revealed a number of peaks with good absorption values because of their multiple proposal design. Moreover, the Q factor and FOM are also used to assess sensor performance. One of the neurological problems that can be treated with cerebrospinal fluid is brain tumors, which can help identify malignant conditions. An MTM-based structure in the THz region can be used to detect brain cancer^23^. This investigation revealed a single peak for their structure and tested the sensor for various RIs; the sensitivity value was 0.502 THz/RIU. Since brain tumors pose a number of challenges for medical imaging, a mtm sensor using several metamaterials has been presented^24^ for early diagnosis. In this investigation, the sensitivity was 1538 GHz/RIU, and the q factor was 19.586 RIU$^{-1}$. They also worked with a regression model because machine learning is capable of optimizing the structure. Proposed^25^ a six-band mtm structure with machine learning support for biomedical applications. Tests of their sensor performance for a single band revealed excellent sensitivity and q factor values, various types of absorption values are discovered. Their regression models indicated that using machine learning to anticipate the design structure might save 60% of the time. A polyimide film-based mtm absorber was introduced^26^ for sensing purposes. This study’s sensitivity value is 1.4 THz/RIU, and it discovered multiple absorption peaks with low and high absorption values. Additionally, this study uses machine learning algorithms to improve the accuracy of the design parameter predictions. They used a variety of test cases in the regression analysis and discovered that the model can identify the optimal structure in as little as 60% of the time. For sensing and gas detection, a multiband mtm absorber in the THz region has been suggested^27^. They discovered multiple peaks and an absorption value of over 90%. They then used machine learning to forecast the design structure and concluded that their approach could reduce the simulation time by 60%.

Many previous studies have proposed various types of sensors for biomedical sensing and brain tumor detection. However, these approaches often face limitations such as low sensitivity, reduced absorption during sensing tests, and resonance peak deviations when the sensing layer is implemented. Moreover, many studies analyze single-band sensitivity, whereas multichannel sensors provide improved accuracy and reduced errors across multiple frequency bands, they also enable simultaneous detection through different channels^28,29^.

Our aim is to develop a unique terahertz metamaterial structure that addresses the limitations of previous sensors while providing high overall performance. To achieve this, a novel and compact absorber with an overall dimension of $35 \times 35 \times 3.8 \upmu m^3$ is proposed for the detection of early-stage neurological disorders. The design is kept simple to ensure a precise fabrication process with minimal potential losses, while the structural novelty arises from the hybrid configuration, including the sizes and placement of the elements, which combines an outer square resonator with inner concentric hexagonal rings and strategically placed gaps. This design enables multiple resonant pathways and strong electromagnetic field confinement at the resonance frequencies. Consequently, the proposed absorber achieves absorption exceeding 99% at all resonance peaks, along with high sensitivity ($\ge$ 1.5 THz/RIU) and favorable figure of merit (FOM) and Q-factor values, demonstrating its reliability for sensing applications. Further we also analyzing the electric field, magnetic field, and surface current distributions to understand the underlying physical mechanisms. To validate the design, an equivalent model is created in ADS software and compared with CST simulation results, and the sensor is tested on different brain tissues, demonstrating the capability to differentiate between various conditions. To enhance design efficiency, several machine learning models are employed to optimize and predict the structure, with Gradient Boosting achieving high accuracy for TC-20 and strong performance for TC-60, reducing simulation time by up to 60%. To enhance transparency and interpretability of the machine learning predictions, explainable AI techniques such as SHAP and LIME are employed. These methods identify the contribution of each design parameter to the sensor performance, providing insight into the underlying factors governing absorption and resonance behavior, which is rarely addressed in metamaterial sensing studies. Overall, the compact size, high absorption, multiband sensing capabilities, and integration with ML-based optimization make this sensor a promising candidate for the early diagnosis of neurological disorders.

## Design methods and analysis of MMA

**Fig. 1:**
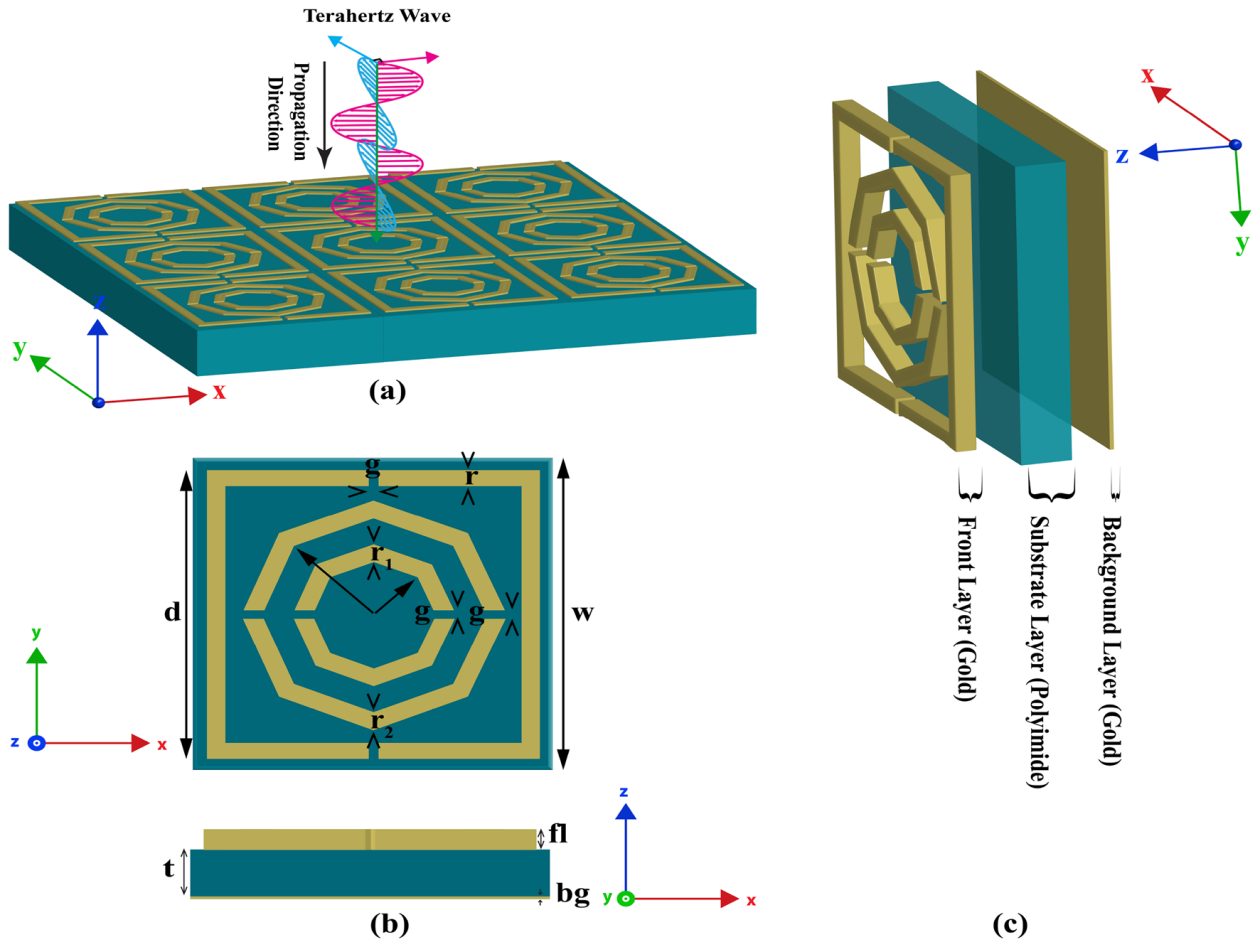
The diagram of the proposed MMA (**a**) periodic array, (**b**) top and side view, (**c**) 3D view.

Our primary goal in the design was to create a simple structure with a high absorption value and good sensing capabilities. For design and simulation purposes, we utilize the CST Microwave Studio software. The materials used in this layout are common and have been widely used in recent studies, and we employ them because they provide optimal performance. We employ a 0.2 $\upmu m$ gold layer on the rear of our construction to block any transmission and help us achieve the highest absorption value. The front size also uses a layer of gold, which has a density of 19,320 kg/m$^3$, a Poisson’s ratio of 0.42, and a heat capacity of 0.13 J/K/kg. The polyimide, sandwiched between two gold layers, has several key characteristics, including a thermal conductivity of 0.2 W/K/m, a Poisson’s ratio of 0.4, and an epsilon value of 3.5. The substrate measures t = 2.5 $\upmu m$, the top layer is fl = 1.1 $\upmu m$, the bottom layer is bg = 0.2 $\upmu m$, and the size of the entire structure is w = 35 $\upmu m$. The front layer features two octagonal shapes cut with the same value, as well as a square shape cut with a one $\upmu m$ gap. The structure can be designed in several ways, such as by first creating a layer with the highest value and then cutting it by developing the same structure with the lowest value. However, this stage is simplified by the CST cylinder option, which offers two options for the entire task: the outer radius and the inner radius, along with segments that specify the type of construction. We utilise values of 23 and 20.5 $\upmu m$ for the square design, represented by r1, and the segment value is 4. This automatically generates a lovely, d = 32.5 $\upmu m$ square structure, which is then rotated 45 degrees to obtain the design structure. Similarly, for the two octagonal designs, 13 and 11 $\upmu m$ are used for the second structure mark (r2), and 8 and 6 $\upmu m$ are used for the last small structure (r3). The segments for these shapes use 8, which gives the octagonal shape exactly. Figure 1(b) provides a detailed look at these factors.

Figure 1 illustrates the metamaterial structure from various perspectives. The periodic array structure and the interaction between the THz wave and the structure are depicted in Fig. 1a. For this simulation, we use a unit cell in both the x and y directions and add open space in the z direction. The wave hits this structure in the z direction, and its angle is 0 in terms of z. Additionally, Fig. 1c displays this structure in three dimensions, making it easy to see how it was constructed layer by layer. Here, we also discuss the materials used in various structures.

**Fig. 2:**
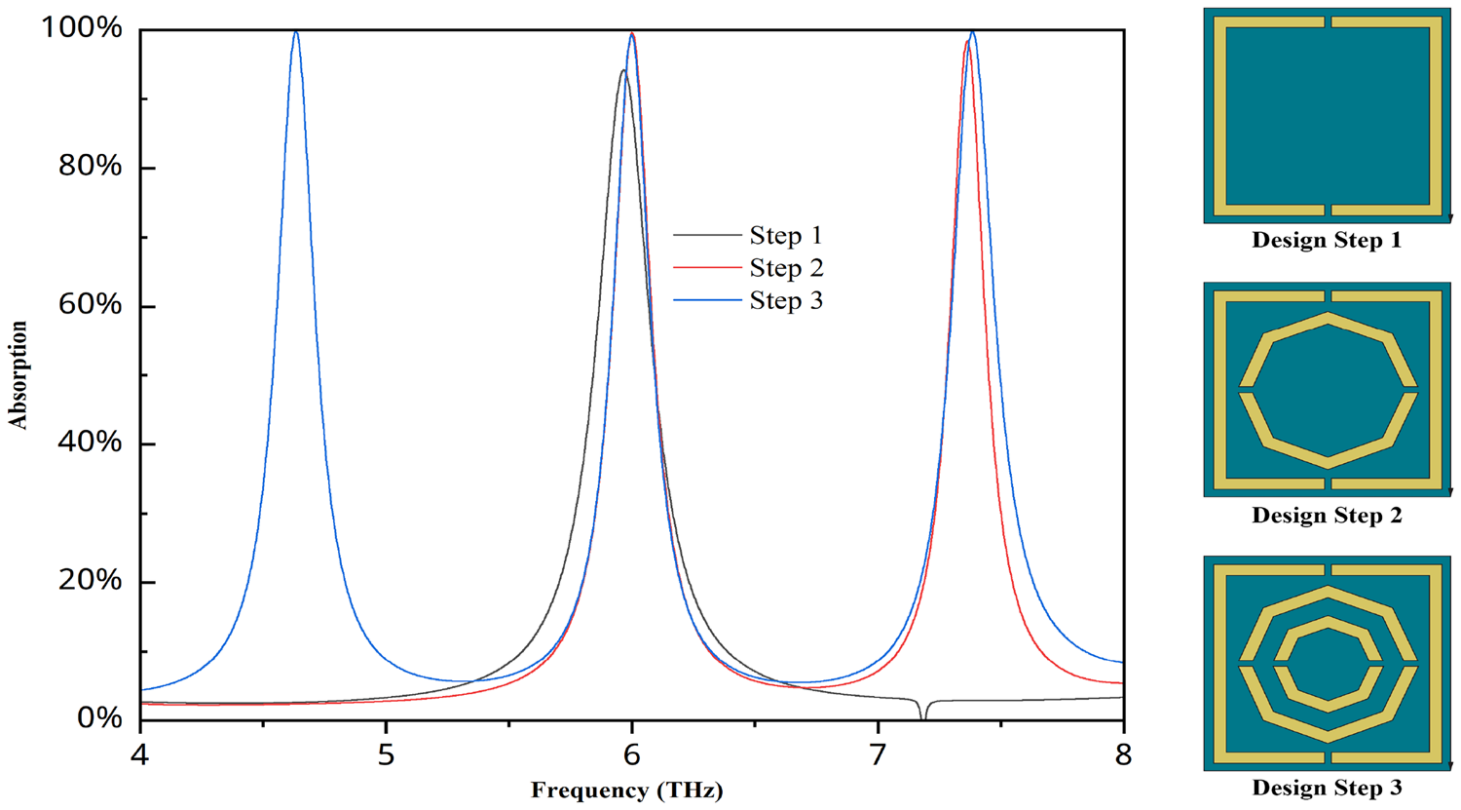
Step-by-step design and results for this proposed structure.

The layout’s step-by-step design is illustrated in Fig. 2, where step 1 involves creating the rectangle. Initially, there is only one graph displayed for the absorption value of this structure. Inside this structure, an additional octagon was built, resulting in two absorption graphs with high absorption values. Our primary goal was to work with a triple band, as it enables multichannel detection, which improves accuracy while operating over an extensive frequency range. After adding the second octagonal shape, we obtained a triple band with a high absorption value.

**Fig. 3:**
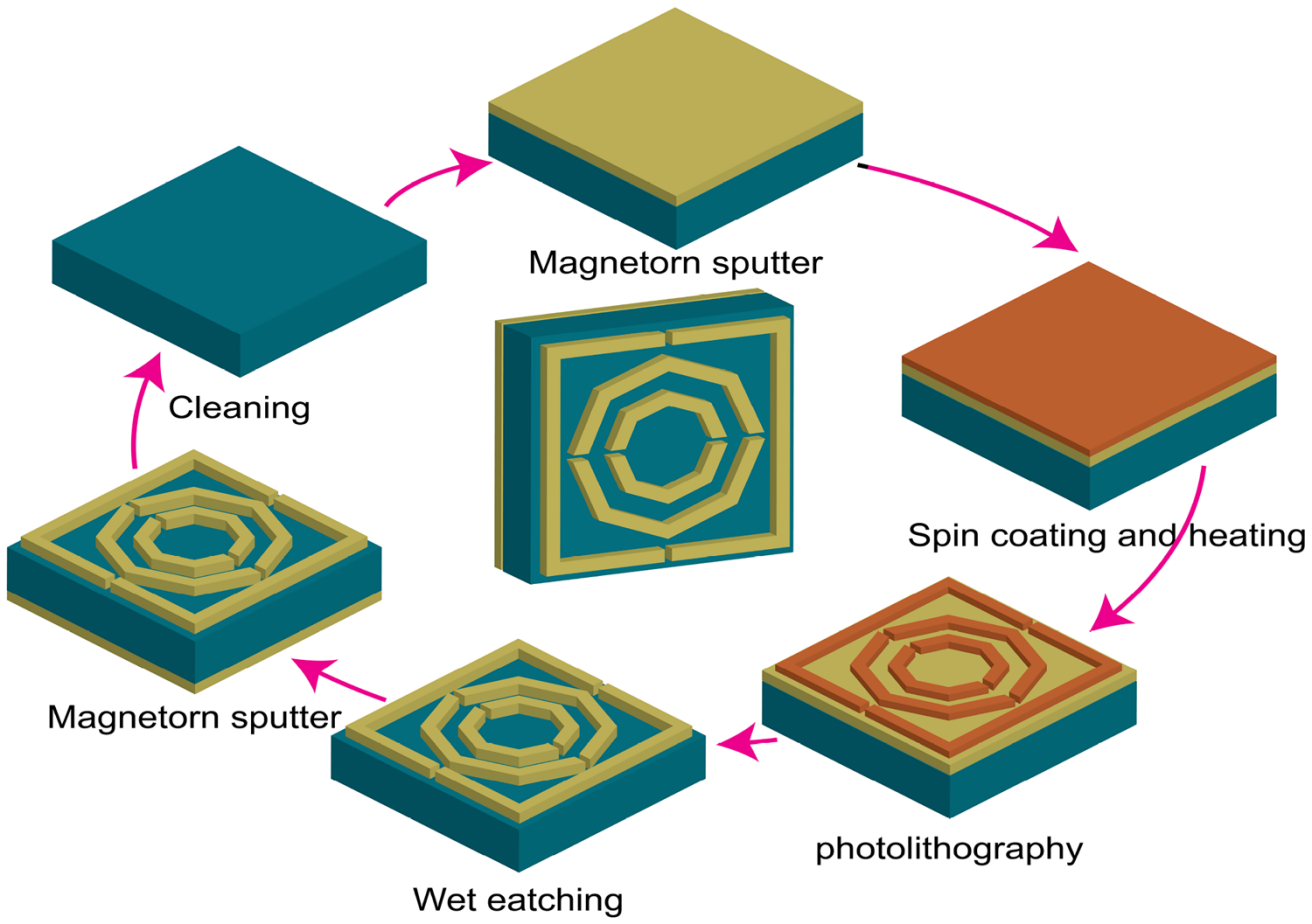
Possible fabrication process for this structure.

Fabrication is essential for this structure because of its practical applications. Since this is a thin structure, fabrication may present some challenges. Nevertheless, this structure can be deposited here via sophisticated production techniques. This structure can be made via photolithography and the wet etching process^30^. The possible fabrication process for this structure is shown in Fig. 3. In the fabrication procedure, the structure is first cleaned, and the front layer of gold is deposited using the magnetron sputtering method. Spin coating, heating, and photolithography are then employed to pattern the front structure accurately. Wet etching is subsequently performed to finalize the front layer. Finally, the back layer is fabricated using magnetron sputtering, completing the overall fabrication of the structure. This structure can also be fabricated via Inkjet printing, which is quicker, more flexible, and less expensive than traditional methods^31^.

The main limitations of metamaterial fabrication include controlling feature sizes at the micro- and nanoscale, achieving uniform thickness across the substrate, and avoiding defects during layer deposition. Producing intricate patterns and aligning multiple layers precisely can also be challenging. Moreover, material stress and thermal expansion during fabrication may affect performance^32^. However, these issues can be mitigated using the discussed fabrication methods, which help reduce losses. In addition, the simplicity of the proposed design further minimizes loss and makes the structure easier to manufacture reliably.

### Absorption analysis

Absorption is a crucial metric for determining how much light a structure can absorb, and metamaterials are particularly well-suited for this purpose, as they enable maximum absorption through design modification. The formula listed below can be used to calculate the absorption value.

1 $$\begin{aligned} A(\omega ) = 1 - R(\omega ) - T(\omega ) = 1 - |S_{11}|^2 - |S_{21}|^2 \end{aligned}$$

The equation states that $R(\omega )$ can be denoted by $|S_{11}|^2$ and that $T(\omega )$ can be $|S_{21}|^2$. Because we use a ground plane of gold, which makes the transmission equal to zero, this helps achieve the maximum absorption value for this structure. Therefore, $T(\omega ) = |S_{21}|^2$ becomes 0 and the Eq. (1) becomes,

2 $$\begin{aligned} A(\omega ) = 1 - R(\omega )= 1 - |S_{11}|^2 \end{aligned}$$

**Fig. 4:**
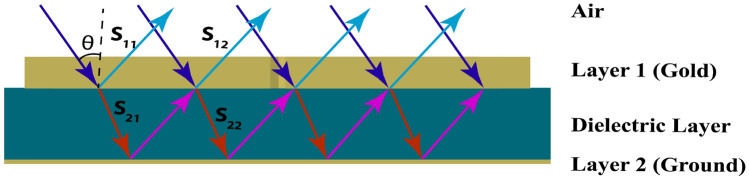
Structure of interference theory model with incident, reflected, and transmitted wave.

According to interference theory, the absorption behavior of this structure can be interpreted as described in^19^. Initially, the top metallic layer is assumed to be absent shows in Fig. 4. The system is then considered as two regions: Region 1 (air) and Region 2 (substrate). The reflection coefficient at the interface from air to Layer 1 is denoted as $S_{11} = |S_{11}|e^{j \theta _{11}}$. The transmission coefficient from Layer 1 from Region 1 to Region 2 is expressed as $S_{21} = |S_{21}|e^{j \theta _{21}}$, The transmission coefficient of Layer 1 from Region 2 to Region 1 is expressed as $S_{12} = |S_{12}|e^{j \theta _{12}}$, and the reflection coefficient in Layer 1 from Region 2 to Region 2 or dielectric to dielectric is given by $S_{22} = |S_{22}|e^{j \theta _{22}}$. Using these parameters, the overall reflection coefficient at the top layer can be calculated as described in^33^.

3 $$\begin{aligned} \sum S_{11} = |S_{11}| e^{j\theta _{11}} + \frac{ |S_{12}|^2 e^{j(\theta _{12} + \theta _{21} - 2\beta - \pi )} }{ 1 - |S_{22}| e^{j(\theta _{22} + \theta _{21} - 2\beta - \pi )} } \end{aligned}$$

Here, $b = kd$, where k represents the wavenumber in the polyimide layer and d is the distance traveled by the transmitted wave within layer 1. The term *b* corresponds to the propagation phase. Owing to the presence of the metallic ground plane, the transmission becomes negligible (approaching zero), and as a result, absorption primarily depends on the reflection parameters. Therefore, Eq. (2) is used to calculate the absorption. Additionally, the space charge equation is employed to further explain the observed absorption phenomena^34^.

4 $$\begin{aligned} A(\omega ) = 1 - R(\omega )= 1 - |S_{11}|^2=\frac{Z_w - n_0}{Z_w+n_0} \end{aligned}$$

To achieve maximal absorption, a material’s wave impedance ($Z_w$ ) must coincide with the free space wave impedance ($n_0$). The material’s impedance is essentially fully resistive and perfectly aligned with the impedance of free space when the real portion of $Z_w$ is one and the imaginary component is approximately zero. This maximizes energy transfer and minimises reflections. The reflection coefficient equation is expressed as $R=| \frac{Z-Z_0}{Z+Z_0}|^2$^35^. When the impedance of medium $Z=\sqrt{\upmu /\epsilon }$ equals that of its surrounding medium $Z_0=\sqrt{\upmu _0/ \epsilon _0}$, the reflections become 0, and the absorption value reaches its maximum position as the equation becomes $A(\omega ) = 1$.

**Fig. 5:**
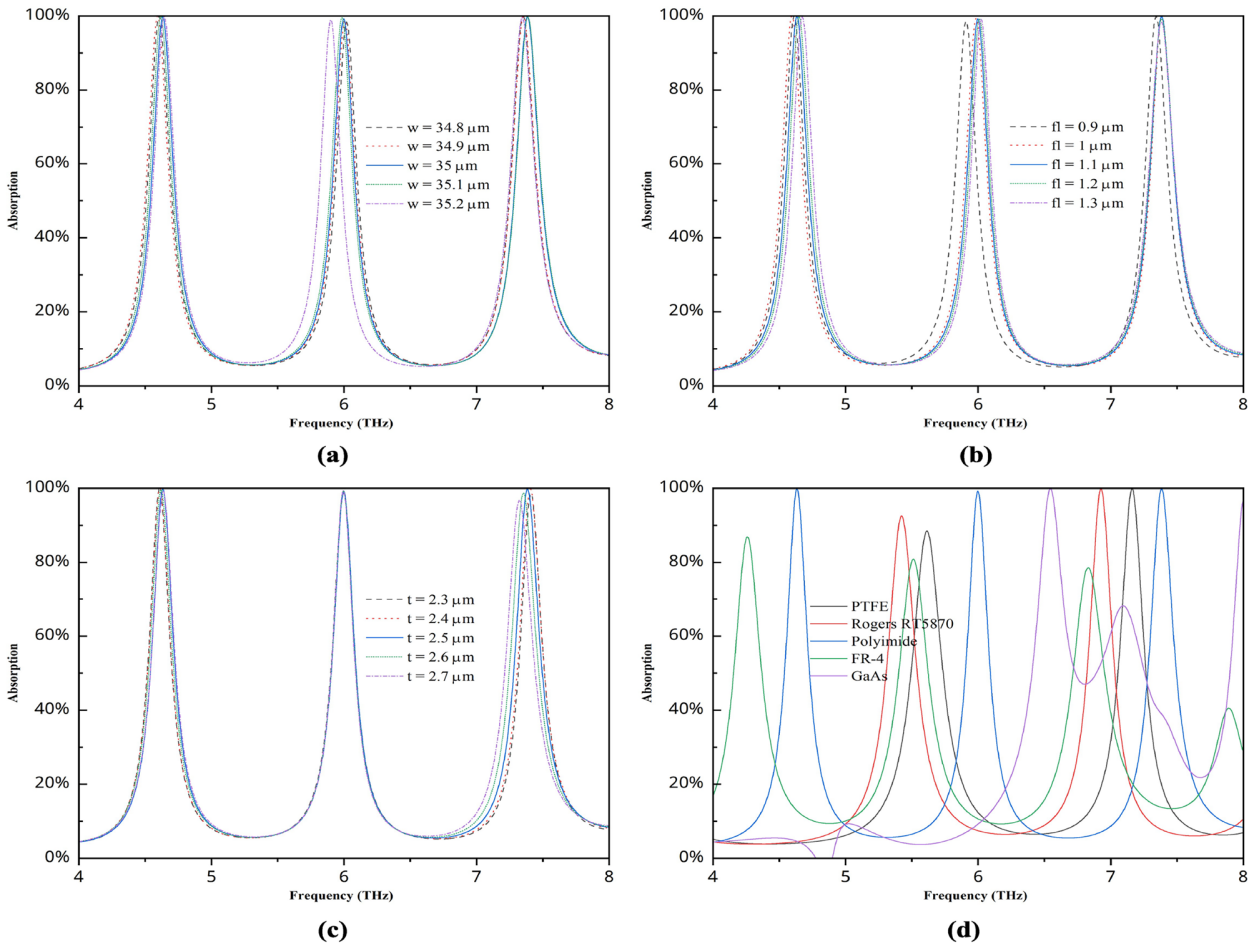
Absorption characteristics for different (**a**) widths and heights, (**b**) thicknesses of the front gold layer, (**c**) substrate widths, and (**d**) substrate materials.

To better understand the absorption characteristics and evaluate the best-suited structure for this study, we also analyzed several parametric studies. Figure 5 shows the graph for different types of studies that help find the best structure, which yields the overall best performance for this research.

Figure 5a illustrates how changing the width and height of the structure from 34.8 to 35.2 $\upmu m$ results in different graph values while keeping all other graph values the same. Since there are no significant changes in the graph, a variety of widths can be employed if necessary. The ideal width for this investigation is 35 $\upmu m$. Additionally, the graph varies slightly depending on the thickness of the various front layers, and occasionally, some bands produce better results. On the other hand, 1.1 $\upmu m$ provides the triple band with an absorption value of over 99% and aids in improving sensitivity, which we will examine later. The centre band remains constant for varying substrate widths; however, the first and third bands exhibit slight variations. A substrate width of 2.5 $\upmu m$ yields the best results for this investigation. Finally, this study evaluated the effectiveness of various substrates commonly used in metamaterial-based research. The Rogers RT5870 performs marginally better than the two bands that PTFE provides, which have high and average absorption levels. Furthermore, FR-4 has three bands, although its GaAs performance is the worst, and its absorption value is lower. We selected this substrate for the study because the polyimide provides a three-frequency band with a high absorption value.

**Fig. 6:**
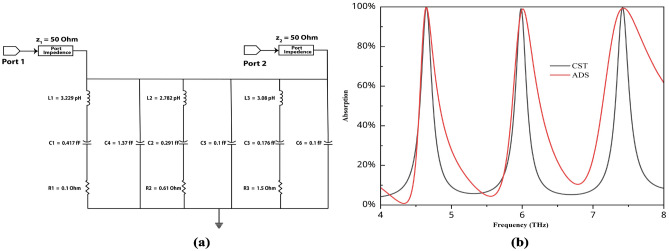
(**a**) Equivalent circuit for this structure, (**b**) ADS vs CST result.

Figure 6a shows the equivalent circuit for this structure. To develop an equivalent circuit, we convert the resonator of the front gold structure into an inductor and split the two microstrip lines with gaps to form a capacitor. The circuit is tuned via ADS software, and the final values are presented in the figure. Figure 6b shows a comparison of the absorption values obtained from both CST and ADS. The peak points remain in the same positions, confirming the high accuracy of the equivalent circuit. We observed that the peaks appeared at the same positions as those in our simulation results, with slight differences in other values. This variation is due to CST producing static results, whereas ADS requires tuning for accurate equivalent circuit modeling.

## Results and discussion

**Fig. 7:**
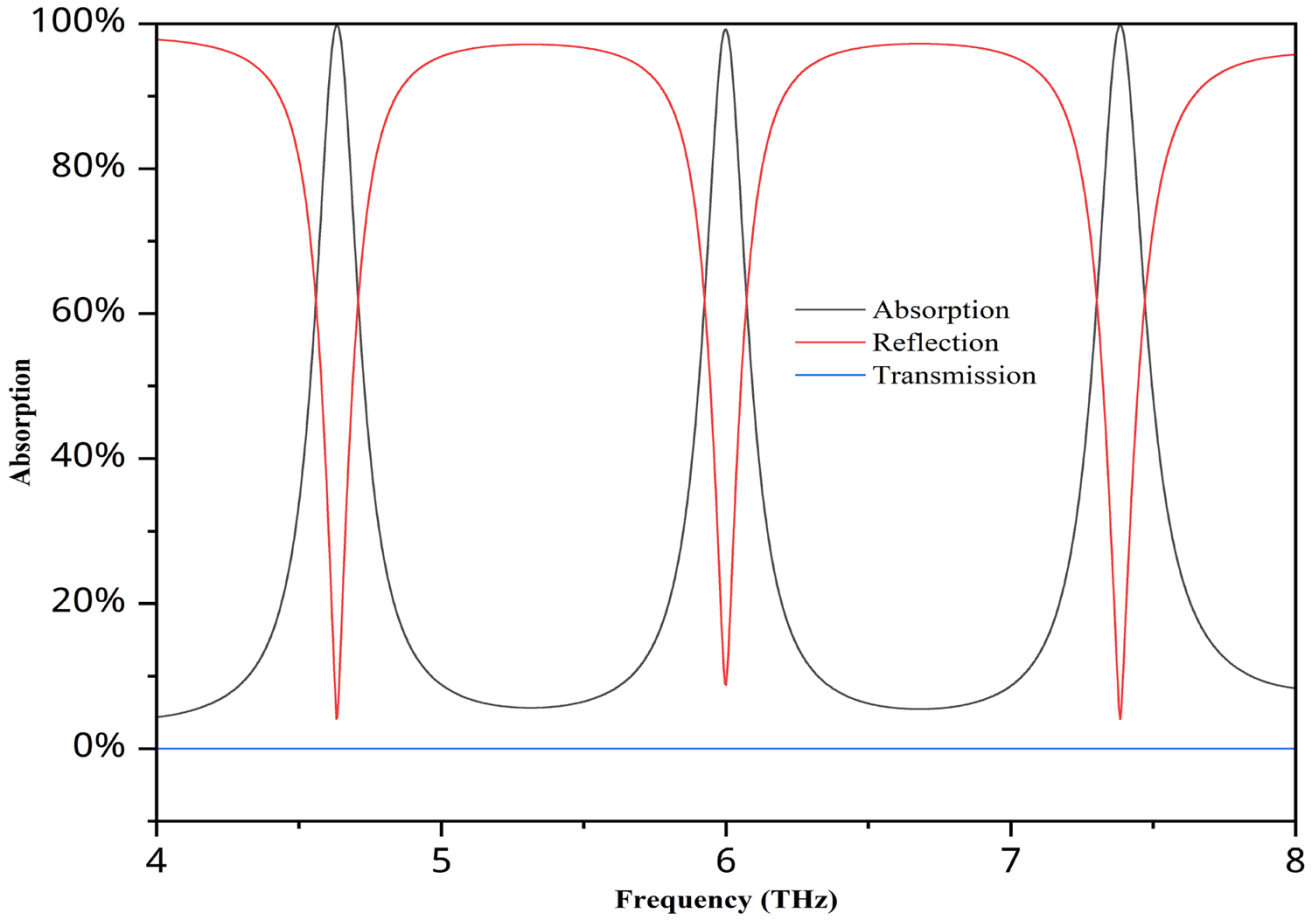
Absorption, reflection, and transmission for this MMA.

The ultimate absorption values for the parameters used in this investigation, as well as reflection and transmission, are shown in Fig. 7. For frequencies of 4.632, 6, and 7.384, all the absorption values were above 99%, specifically 99.8%, 99.3%, and 99.8%, respectively. The transmission value is zero due to the rear layer, which helps achieve the highest absorption value. We utilise Origin Pro, which offers various types of graph analysis, to plot the graph. The breadth of a peak at 50% of its highest height is known as the full width at half maximum (FWHM). The difference between the lower and upper frequency (or value) points at half of the peak’s greatest height can be computed manually or with software such as OriginPro. We used Origin Pro for this investigation, and for three frequency peaks, we obtained fwhm values of 0.20853, 0.20399, and 0.21836. The quality factor (Q-factor) is a vital parameter used to assess the sharpness and selectivity of resonance modes. A higher Q-factor signifies a narrower and more distinct resonance peak, which directly correlates with improved performance and precision in sensing applications. It reflects the device’s ability to detect small changes with high accuracy^25^. The formula used to determine the Q factor is $Q = fc/fwhm$, where *fc* represents the frequency peak and *fwhm* is calculated beforehand. The corresponding q-factor values for these frequencies are 22.21, 29.41, and 33.81. Table 1 compares the absorption values obtained in this study with those of recently proposed works. The absorption value for this sensor is excellent compared with that of similar works, especially its multi-band performance, with an absorption value above 99%.

**Table 1:** Comparison of the absorption with those of previous works.

Ref.	Resonance frequency (THz)	Structure size ($\upmu m$)	Absorption (%)
^36^	0–3	80 $\times$ 80	99, 80, 95
^37^	0.5–4.5	30 $\times$ 30	99, 98, 99
^38^	1–3	-	97, 98, 99
^39^	7–9.5	25 $\times$ 25	98
^40^	4.59, 6.18	2.4 $\times$ 2.4	99.9, 99.3
^41^	2.638, 5.158	30 $\times$ 30	Almost 90
Proposed	4.632, 6, 7.384	35 $\times$ 35	99.8, 99.3, 99.8

**Fig. 8:**
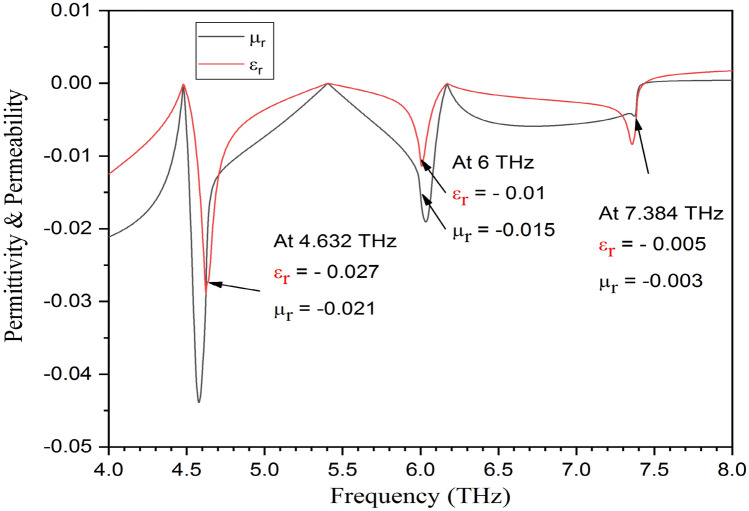
Permittivity & permeability vs Frequency values at 4.632 THz, 6 THz, and 7.384 THz.

The permittivity and permeability both exhibit negative values at the resonance frequencies of 4.632 THz, 6 THz, and 7.384 THz. Figure 8 presents the value of left-handed metamaterial.

### Angle stability analysis

**Fig. 9:**
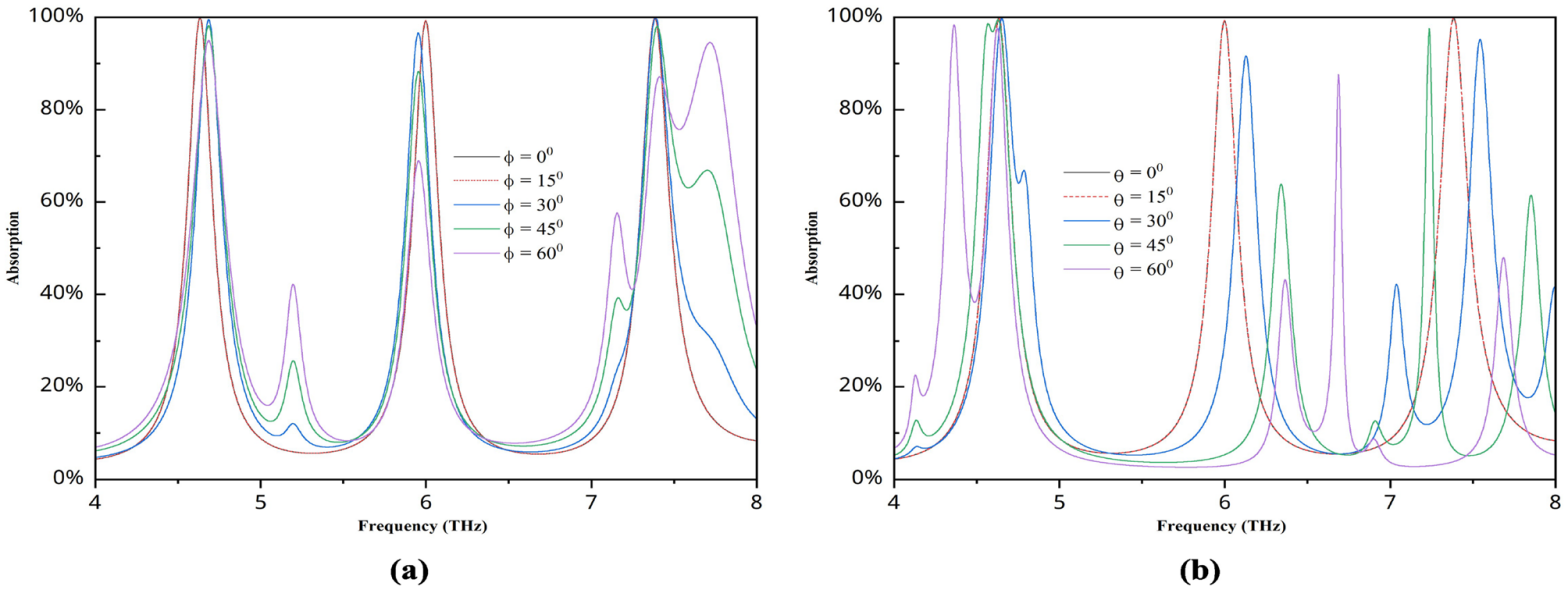
Absorption characteristics for different (**a**) polarization angles, (**b**) incident angles.

The light intersects the structure at a particular angle. Both the incidence angle and the polarization angle are set to zero for the duration of the simulation. We also assess this structure’s performance for varying incident angles and polarizations. The graph is shown in Fig. 9, and it is evident that the angles for 0$^\circ$ and 15$^\circ$ remain constant. Beyond that, it is not maintained; at a polarization angle of 30 degrees, the peak essentially stays the same, but the absorption value decreases. The deterioration is still visible in the graphs for 45$^\circ$ and 60$^\circ$. For incident angles of 30, 45, and 60 degrees, the graph compresses and multiple new peaks appear. Thus, the optimal range for this structure’s performance is 0 to 15 degrees, which is sufficient.

### E-field, H-field and surface current analysis

Maxwell’s equations can explain the relationships among the E-field, H-field, and surface current, as mentioned below^42^.

5 $$\begin{aligned} & \nabla \times H = J + \epsilon \frac{\delta E}{\delta t} \end{aligned}$$

6 $$\begin{aligned} & J = \sigma E \end{aligned}$$

**Fig. 10:**
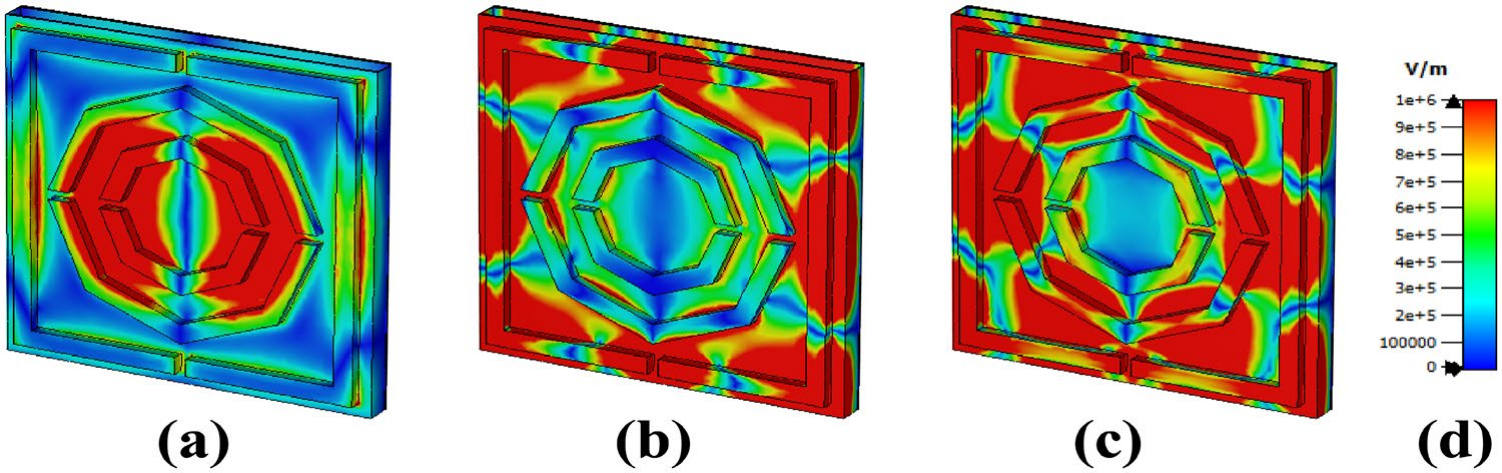
E-field distributions at (**a**) 4.632 THz, (**b**) 6 THz, and (**c**) 7.384 THz.

The red area in the electric and magnetic fields is caused by surface plasmon resonance^43^. The distribution of the E field for our triple-frequency band is displayed in Fig. 10. Due to cavity surface plasmon resonance (CSPR) and localised surface plasmon resonance (LSPR), the left and right sides of the lower octagon and the edge of the second octagonal shape exhibit a high electric field intensity at 4.632 THz^33,44,45^. Furthermore, at 6 THz, a vigorous electric field intensity is observed on both the left and right sides, as well as at the corners of the square shape due to the LSPR, whereas the top and bottom sides show the CSPR. Finally, the last band square shows high intensity and is almost identical to the previous band. The middle layer combines the left and right sides into a square shape due to the CSPR.

**Fig. 11:**
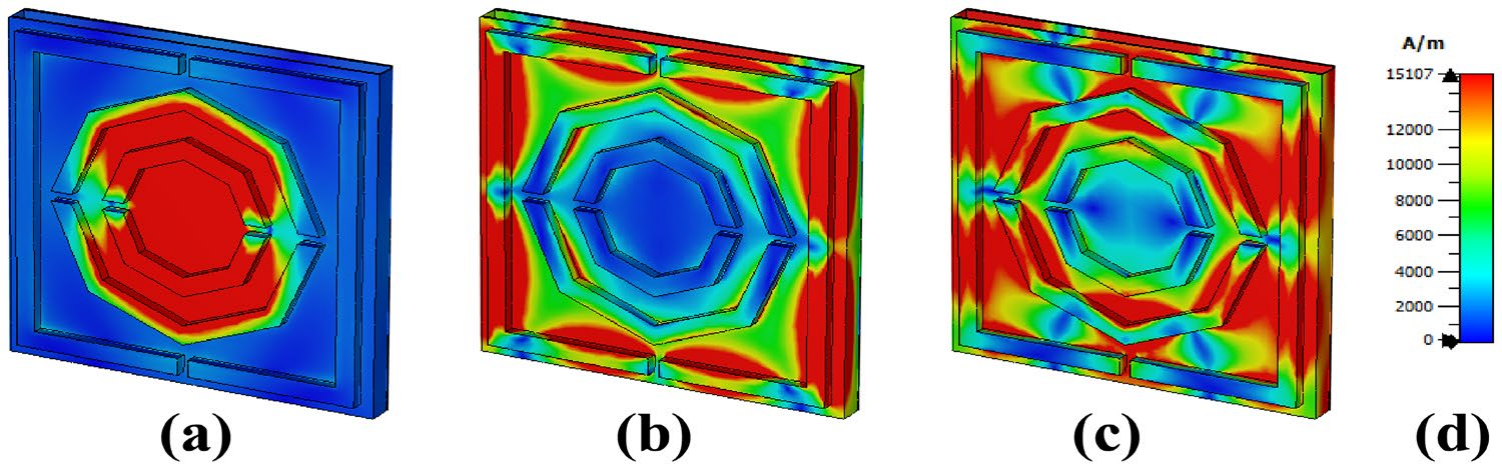
H-field distributions at (**a**) 4.632 THz, (**b**) 6 THz, and (**c**) 7.384 THz.

Owing to the CSPR, the magnetic field has a high strength in the bottom band just for the middle structure and the edge of the second octagonal, as shown in Fig. 11. Due to the LSPR, a high intensity h field is only observed in a square shape on the left and right sides for the 6 THz frequency range. For the higher band magnetic field, high intensity is observed on the left and right sides of the square form, and it merges with the middle layer via the CSPR. The split gap in the octagon differentiates the magnetic field intensity on both the upper and lower sides.

**Fig. 12:**
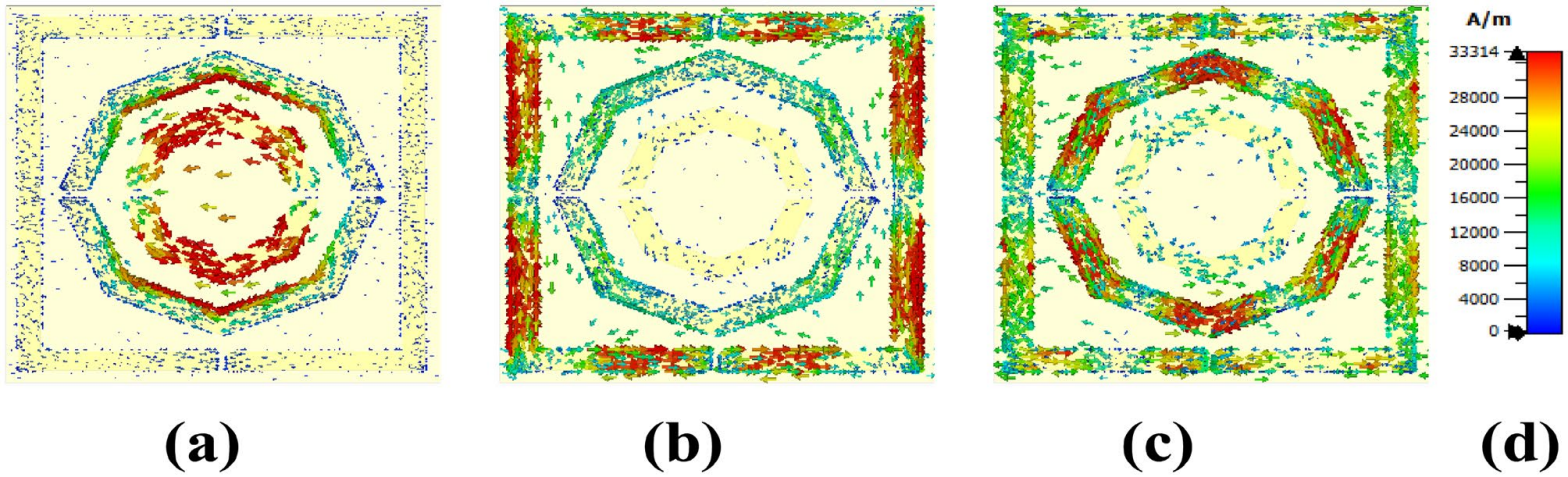
Surface current distribution at (**a**) 4.632 THz, (**b**) 6 THz, and (**c**) 7.384 THz.

The strong localised current was observed where we discovered the strong magnetic field, as illustrated in Fig. 12. For the lower frequency of 4.63 THz, the top and bottom sides of the small octagon exhibited a higher concentration of surface current, as did the bottom edge of the second octagon. For 6 THz, the square form displays a high intensity of strong surface current in several of its areas. There are multiple antiparallel current flows at the bottom of its sides, indicating a large magnetic field, as parallel and antiparallel currents typically indicate a strong magnetic field^46^. At 7 384 THz, the middle octagonal region has a greater concentration of localised current in several of its sections, in addition to its square shape.

### Sensing performance analysis

**Fig. 13:**
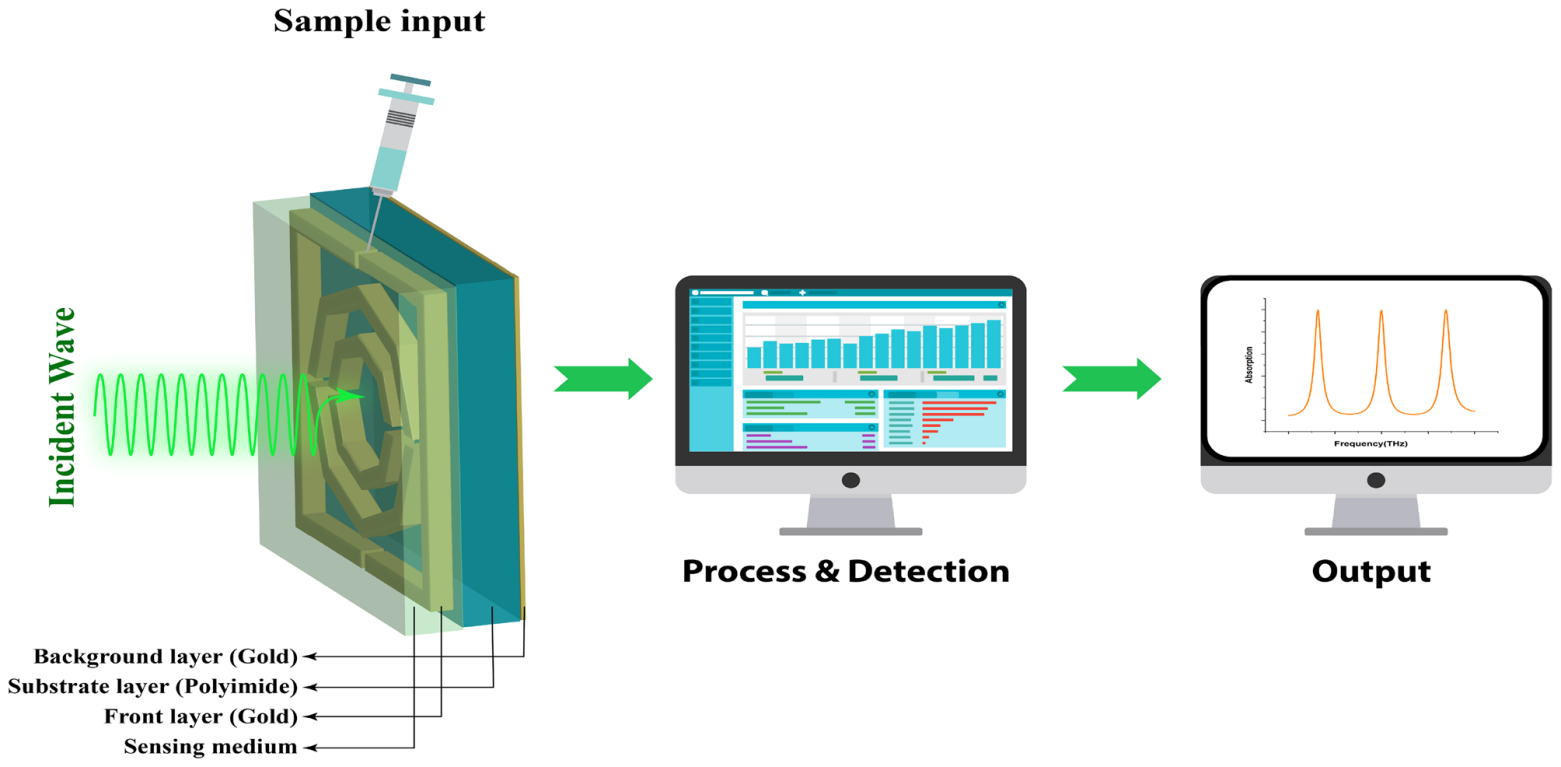
Sensor setup for this absorber.

Figure 13 shows the setup of the sensor for this absorber. Here, a 3 $\upmu m$ analytic layer is added above the substrate. The front gold layer is inside the sensor, and first, it is empty. Then, the biological samples, in liquid form, are placed into the sensor. When the THz incident wave interacts with this layer, the results should show different graphs for different types of values. It will process and detect further, leading to the output result. The variation in the output results helps us identify the condition of the biological samples.

**Fig. 14:**
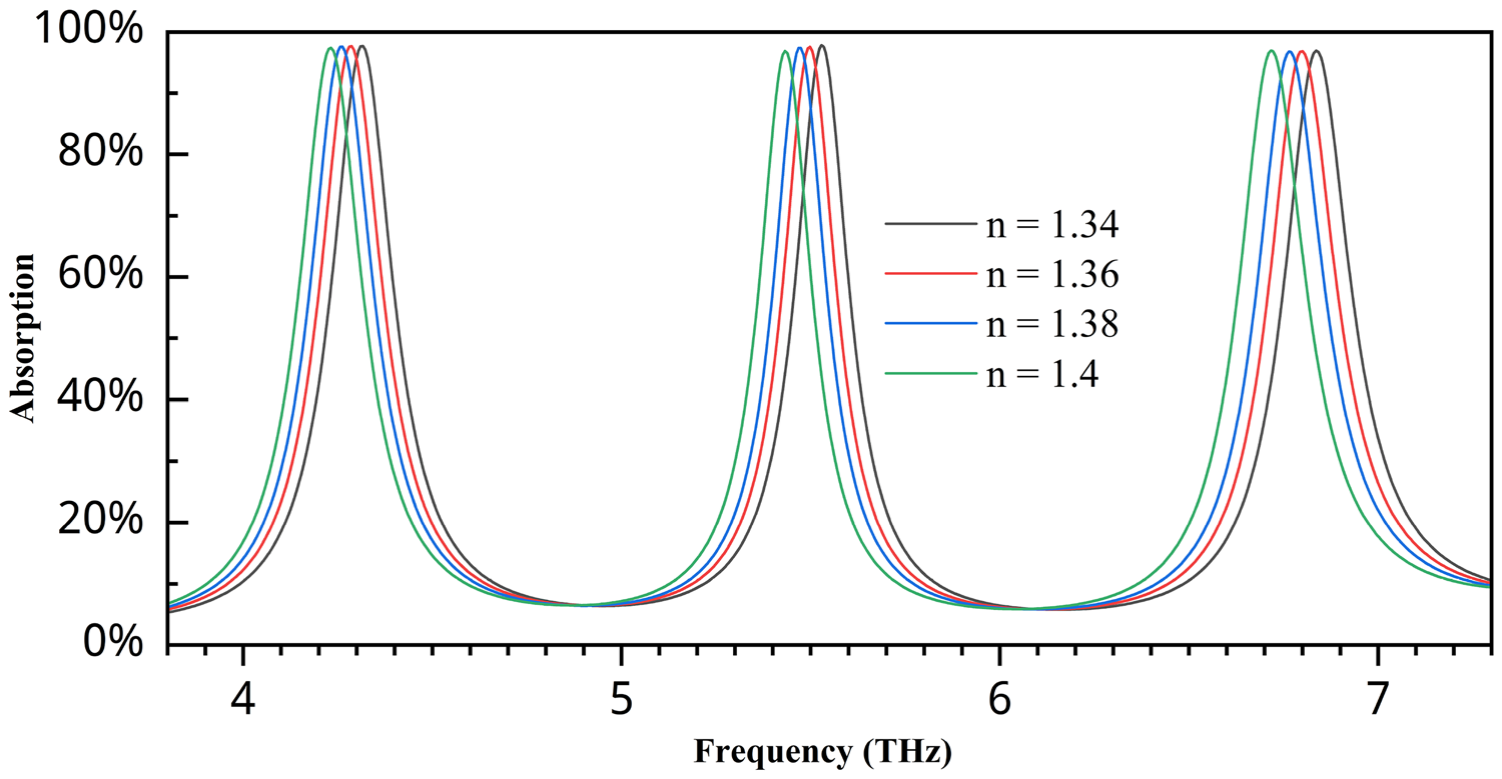
Absorption variation for different refractive index values.

Sensitivity is a crucial characteristic of a sensor, reflecting its ability to detect and distinguish even slight changes in the permittivity of a sample accurately^47^. A higher sensitivity indicates that the sensor can respond effectively to minimal variations in the material’s dielectric properties, which is essential for precise sensing applications. For this absorber, we first checked the sensing performance in the refractive index range from 1.34 to 1.4, where the maximum number of biological samples fell^48^. Figure 14 shows the variation in the graph for changing the RI value, where it clearly indicates a significant difference between the graph and the third peak, which performs slightly better than the first two. There are no tendencies to reduce the absorption value, and it remains above 96%, even as the variation in the RI value occurs. Only the second band shows a slight downward trend, but it remains above 96% absorption. For a refractive index of 1.34, the peaks are observed at 4.316 THz, 5.528 THz, and 6.836 THz for the three bands. For a refractive index of 1.36, they shift slightly to 4.286 THz, 5.498 THz, and 6.8 THz. The sensitivity is calculated via the following formula:

7 $$\begin{aligned} S = \frac{\Delta f_0}{\Delta n} \end{aligned}$$

Here, $\Delta f_0$ is the variation in frequency, whereas $\Delta n$ is the change in the refractive index. After calculation via the following formula, we find sensitivity values of 1.5 THz/RIU, 1.5 THz/RIU, and 1.8 THz/RIU. This is a major advantage of this sensor, as all the bands produce high sensitivity values. Table 2 presents a comparison between this sensor and recent works in the same field.

**Table 2:** Sensitivity comparison with recent literature.

Ref.	Year published	S (THz/RIU)	Oblique incidence	Publication in	Applications
^18^	2025	0.571, 1.15	Up to 45°	Optics Express	Biomedical sensing
^19^	2023	0.103, 0.095	Up to 45°	Scientific Reports	Microorganisms detection
^10^	2025	0.435, 0.7, 1.29	Up to $\le$ 15°	Plos one	Blood cancer diagnostics
^23^	2024	0.502	Up to 30°	Bio. Phy. & Eng. Express	Brain cancer detection
^46^	2023	0.0515, 0.076	Up to 90°	IEEE Access	Skin Cancer Diagnostics
^49^	2021	0.851	-	Plasmonics	RI biosensor
^50^	2024	0.13, 0.14	-	Photon. Nanostru. Fund. Appl.	sensing
^51^	2023	0.495	-	Optik	Biosensor
^52^	2024	-	Up to 50°	Physica Scripta	Terahertz applications
^53^	2025	0.38, 0.88, 1.9, 1.64, 2.40, 3.67	Up to $\le$ 40°	Mat. Science	Eng. B & RI sensor
This work	2025	1.5, 1.5, 1.8	Up to 30°	-	Early stage neurological disorder detection

The comparison table shows that our proposed sensor’s sensitivity exceeds that of several recent works, indicating its superior accuracy in sensing. Notably, the triple-band results reported by Hamza et al.^10^ show both high and low sensitivity across multiple modes. Similarly, studies such as^46^ and^19^ present dual-band results with relatively lower sensitivity. Though some works show greater sensitivity, our work demonstrates consistent performance across all bands and a wide range of RI values. Moreover, we included the incident angle, where our structure maintains its performance up to 30°, which we believe is sufficient. In contrast, our sensor performs exceptionally well in multi-band sensing, making it an ideal candidate for advanced sensing applications.

The figure of merit (FOM) plays an integral role in assessing and comparing the performance of various sensors in terms of their sensing capabilities^32^. It is calculated using Eq. (8)^48^,

8 $$\begin{aligned} FOM = \frac{s}{FWHM} \end{aligned}$$

In this study, after computing the FWHM for each resonance, the FOM values were found to be 7.19, 7.35, and 8.24 at 4.63 THz, 6 THz, and 7.38 THz, respectively, indicating excellent sensing performance across all channels.

### Neurological disorder detection

**Fig. 15:**
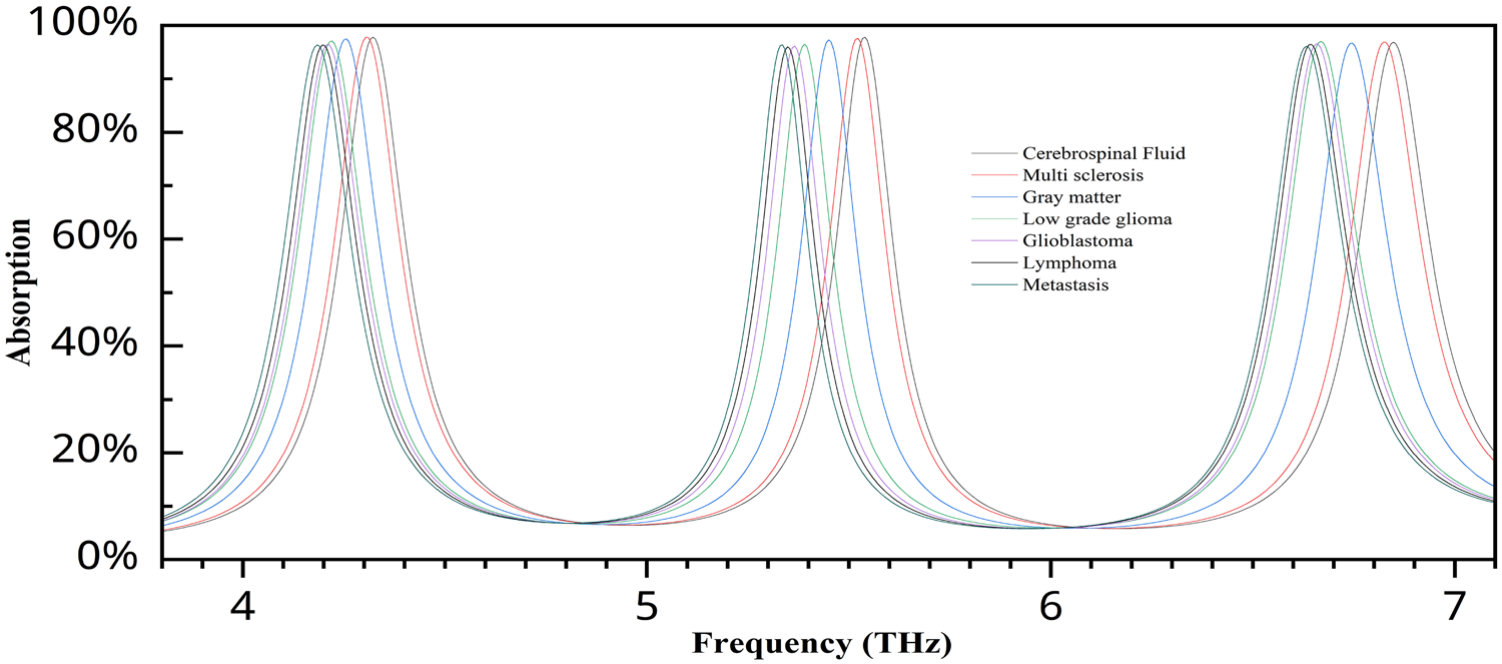
Absorption variation for different brain tissue values.

**Fig. 16:**
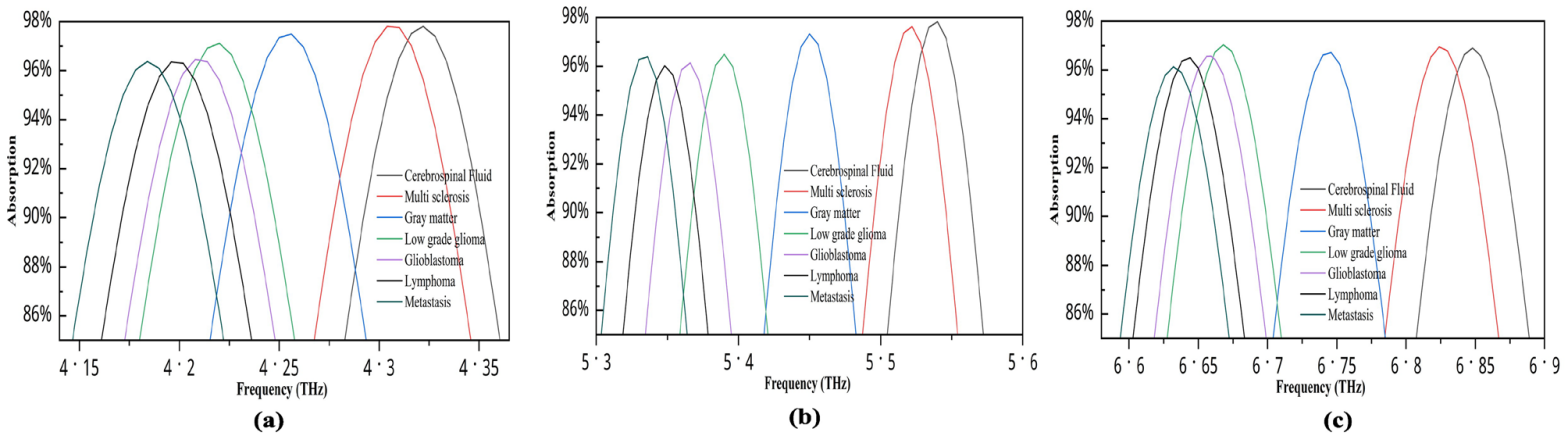
Enlarged view of the absorption variation for different brain tissue values: (**a**) first peak, (**b**) second peak, (**c**) third peak.

This sensor has high sensitivity and can detect even minor variations in the refractive index value, which is helpful in detecting neurological illnesses in their early stages. Furthermore, its triple-mode resonance capacity enables the sensor to function at various frequencies, allowing for multiple detection channels and thereby enhancing accuracy. Table 3 presents the refractive index values of different brain cell conditions^4^, which are subsequently used in the sensing analysis. As shown in Fig. 15, the resonance frequencies shift toward the lower end (redshift) as the refractive index of the brain tissues increases. The distinct peak positions across all three channels show clearly separated the tissue types, indicating strong discriminatory capability. To further highlight this, an enlarged view is presented in Fig. 16, which confirms that there is no significant overlap in the peak points between the spectra of different tissues across any of the sensing channels. These findings demonstrate that the proposed triple-band metamaterial absorber is highly suitable for detecting subtle differences in the refractive indices of brain tissue. Notably, the sensor can analyze variations in the refractive index of cerebrospinal fluid (CSF), which is associated with changes in water content. These changes are often early indicators of neurological disorders. Therefore, the sensor’s ability to distinguish such variations suggests its potential in identifying early-stage neurological conditions through noninvasive refractive index monitoring.

**Table 3:** Refractive index value of several brain conditions.

Brain tissues	Refractive index
Cerebrospinal fluid	1.3333
Multiple sclerosis	1.3425
Gray matter	1.3951
White matter	1.4121
Low-grade glioma	1.432
Glioblastoma	1.447
Lymphoma	1.4591
Metastasis	1.4833

## Machine learning modelling to predict absorption

**Fig. 17:**
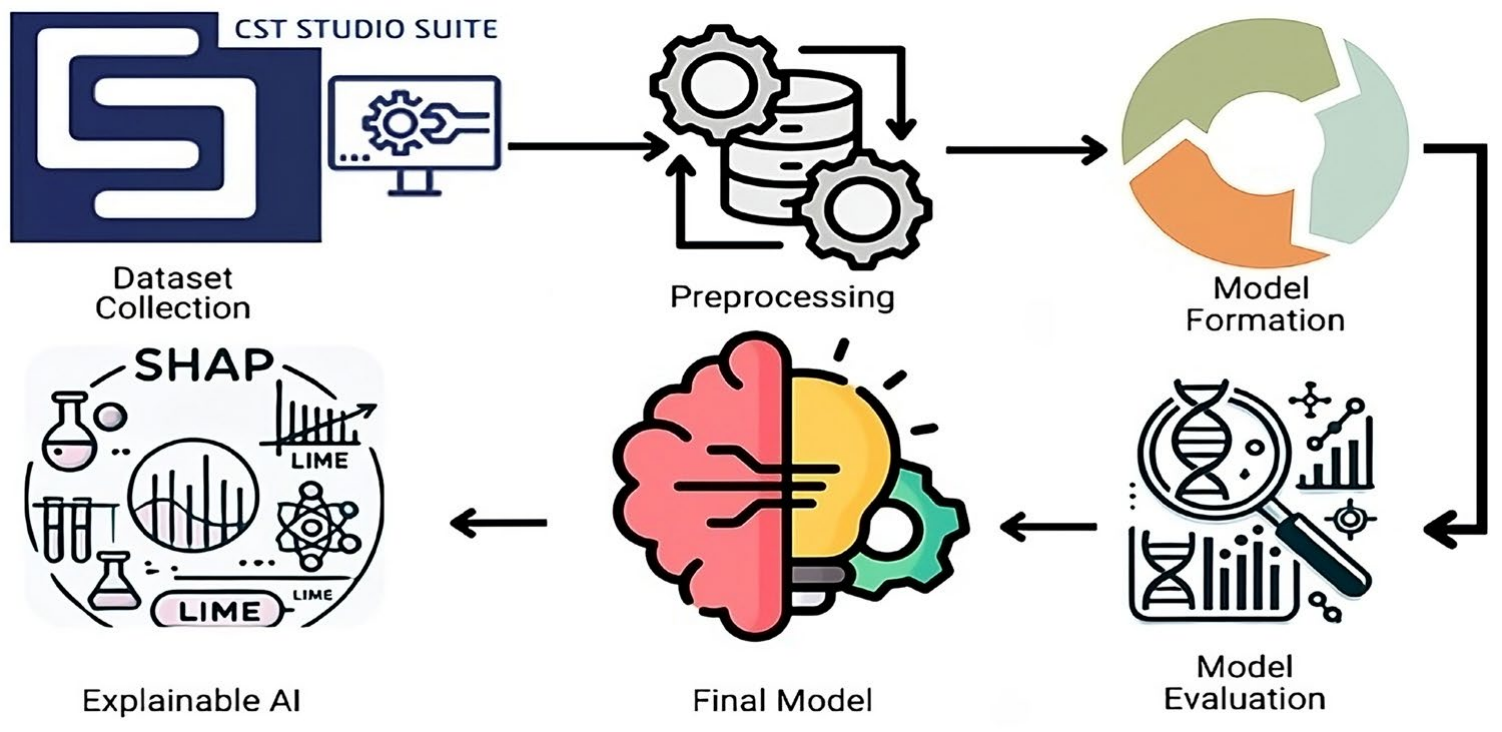
Model architecture.

The simulation of complex absorber designs is notoriously time-consuming and resource-intensive, and often requires exhaustive experimental setups. By employing machine learning regression techniques, such as surrogate or meta-models, it is possible to predict the absorption coefficient from frequency inputs without running full-scale simulations for every configuration. These models learn the underlying input-output relationships and can fill in missing parameter values, dramatically reducing computational demand dramatically by as much as 40–80%. In engineering domains, ML-augmented simulations have delivered speedups ranging from milliseconds to orders of magnitude faster than traditional methods do^54^. This efficiency empowers researchers to explore broader design spaces, iterate rapidly, and generalize to unseen data, thereby transforming a slow and costly process into a fast and intelligent modeling pipeline, without sacrificing accuracy. Figure 17 shows the architecture of the machine learning model.

### Data annotation and preprocessing

The sensor dataset utilized in this study was generated via the CST Microwave Studio simulator, which encompasses 31,710 unique device configurations. These configurations were systematically derived by varying five key design parameters: substrate thickness (2–3 $\upmu m$), resonator thickness (0.50–1.50 $\upmu m$), background layer thickness (0.10–0.30 $\upmu m$), split gap (0.50–1.50 $\upmu m$), and substrate dimension (34–36 $\upmu m$). This approach ensures a comprehensive exploration of the design space, facilitating the identification of optimal configurations for specific sensing applications. The dataset was partitioned into input features and target variables. The input features comprise the aforementioned design parameters, while the target variable is the magnitude of the absorber layer. These datasets are instrumental in training machine learning models to predict the performance characteristics of metamaterial-based sensors. For example, studies have demonstrated the efficacy of machine learning techniques in predicting the resonance frequency shifts of metamaterial sensors based on design parameters. To ensure reliable predictive performance, the data were initially cleaned to remove noise and outliers. A hold-out validation strategy was then applied, with 80% of the data used for training and 20% used for testing. Additional splits—60:40, 50:50, and 40:60—were also evaluated to assess the performance sensitivity to the proportion of training data.

### Model development

In this investigation, various supervised machine learning models were evaluated for prediction, including Gradient Boosting Regression, Extra Trees Regressor , XGBoost (XGB), Random Forest Regressor (RFR), Decision Tree Regressor (DTR), Artificial Neural Network (ANN), and k‑Nearest Neighbors (KNN)^55–61^.

### Hyperparameter optimization

In this study, we employed two well-established methods for hyperparameter tuning, grid search and randomized search, to increase the performance of the selected models and improve their predictive accuracy^62,63^. In grid search, one defines a finite set of hyperparameter values and exhaustively evaluates the model across all possible combinations, selecting the configuration that achieves the best performance. In contrast, randomized search samples hyperparameter configurations randomly from the same predefined search space, significantly reducing the computational cost while often yielding comparable outcomes with fewer evaluations. Hyperparameter optimization can substantially enhance the performance of machine learning models. Nevertheless, these methods are usually computationally intensive and time-consuming, particularly when searching over extensive hyperparameter spaces or tuning complex architectures^64,65^. Machine learning models contain distinct sets of hyperparameters, and cross-validation, a robust sampling verification technique, has been employed to assess the accuracy of each model. This method mitigates overfitting and provides a more generalized performance evaluation. For optimizing model performance, default hyperparameter configurations are utilized as baselines, which can be further refined through systematic tuning approaches, such as grid search or randomized search^66,67^. To validate the results, an alternative optimization technique, Bayesian optimization, is employed. This method distinguishes itself from grid search and random search by enhancing the efficiency of the hyperparameter tuning process. Bayesian optimization constructs a probabilistic model to predict the performance of various hyperparameter configurations. It uses an acquisition function to determine the next set of parameters to evaluate, effectively balancing the exploration of new configurations with the exploitation of known promising configurations. This approach aims to identify the optimal hyperparameters with fewer evaluations, making it particularly advantageous for computationally expensive models^68^.

### Machine learning model evaluation

This section presents an analysis and interpretation of the simulation results. All the experiments were performed in Python via Google Colaboratory. A suite of libraries, including Pandas, NumPy, Matplotlib, Seaborn, Scikit-learn, Keras, SHAP, and LIME, was utilized for data handling, visualization, model development, and explainability. The dataset comprises 31,710 samples generated via CST simulations, with five input features and one output. The performance of the regression models is visualized in Table 4. Among the evaluated models, gradient boosting achieved the highest predictive accuracy (R$^2$ = 0.9978, RMSE = 0.0120, MAE = 0.0052), followed closely by XGBoost (R$^2$ = 0.9963, RMSE = 0.0157, MAE = 0.0075). Both models captures the complex frequency-to-absorption relationships with exceptional precision. The random rorest also demonstrated strong performance (R$^2$ = 0.9901; RMSE = 0.0257; MAE = 0.0101), benefiting from ensemble averaging to reduce overfitting and improve generalizability. Moreover, the decision tree (R$^2$ = 0.9470; RMSE = 0.0594; MAE = 0.0258) provided interpretable results but exhibited signs of overfitting. K-nearest neighbors (KNN), although intuitive and straightforward, yielded the lowest predictive accuracy among the top models (R$^2$ = 0.9240; RMSE = 0.0712; MAE = 0.0244), likely due to its sensitivity to data scale and dimensionality. These findings highlight that ensemble-based boosting algorithms, particularly gradient boosting and XGBoost, are the most suitable for accurately and efficiently predicting absorber performance, thereby minimizing the need for computationally expensive simulations. To validate model stability, a 10-fold cross-validation (Table 5) procedure was applied to the gradient boosting regressor. The average R$^2$ score was 0.9979 (±0.0006), while adjusted R$^2$ exhibited identical consistency. The mean squared error remained extremely low ($\approx$0.0001), with an average RMSE and MAE of 0.0117 (±0.0017) and 0.0049 (±0.0004), respectively. This striking uniformity in performance indicators across folds confirms not only the model’s exceptional accuracy but also its reliability and generalizability. Consequently, the gradient boosting model is validated as a robust and precise predictor of absorber performance, that is capable of closely emulating computationally expensive CST simulations.

**Table 4:** Comparative evaluation of different models.

Model	Types of hyperparameters	R$^2$	Adj R$^2$	MSE	RMSE	MAE
Gradient Boosting	Randomized Search	0.9978338	0.997831	0.001532	0.039140	0.018789
	Grid Search	0.976996	0.976970	0.001532	0.039140	0.018789
	Bayesian Optimization	0.997467	0.997465	0.000169	0.012987	0.005995
XGBoost	Randomized Search	0.996285	0.996281	0.005063	0.071156	0.024387
	Grid Search	0.923969	0.923885	0.005063	0.071156	0.024387
	Bayesian Optimization	0.996375	0.996371	0.000241	0.015537	0.007651
RandomForest	Randomized Search	0.990081	0.990070	0.005639	0.075092	0.036513
	Grid Search	0.915325	0.915232	0.005639	0.085153	0.036513
	Bayesian Optimization	0.991450	0.991440	0.000569	0.023862	0.009058
ExtraTrees	Randomized Search	0.971125	0.971093	0.007251	0.085153	0.042693
	Grid Search	0.891116	0.890995	0.007251	0.093346	0.042693
	Bayesian Optimization	0.976291	0.976264	0.001579	0.039736	0.012471
DecisionTree	Randomized Search	0.947020	0.946961	0.008713	0.093346	0.049031
	Grid Search	0.869157	0.869013	0.008713	0.106051	0.049031
	Bayesian Optimization	0.969810	0.969777	0.002010	0.044839	0.018139
KNN	Randomized Search	0.923969	0.923885	0.011247	a0.106051	0.056097
	Grid Search	0.831116	0.830929	0.011247	0.113157	0.056097
	Bayesian Optimization	0.923969	0.923885	0.005063	0.071156	0.024387
ANN (128-64)	Randomized Search	0.777013	0.776767	0.012805	0.113157	0.062361
	Grid Search	0.807725	0.807512	0.012805	0.236930	0.059858
	Bayesian Optimization	0.788194	0.787959	0.014105	0.118765	0.063834
AdaBoost	Randomized Search	0.167448	0.166528	0.056136	0.236930	0.196193
	Grid Search	0.157050	0.156118	0.056136	0.196697	0.196697
	Bayesian Optimization	0.203702	0.202822	0.053029	0.230281	0.191788

**Table 5:** 10-Fold cross-validation results for gradient boosting.

Fold	R$^2$	Adj R$^2$	MSE	RMSE	MAE
1	0.9979	0.9979	0.0001	0.0118	0.0049
2	0.9985	0.9985	0.0001	0.0098	0.0044
3	0.9975	0.9975	0.0002	0.0128	0.0052
4	0.9983	0.9983	0.0001	0.0105	0.0045
5	0.9982	0.9982	0.0001	0.0109	0.0047
6	0.9967	0.9967	0.0002	0.0152	0.0053
7	0.9979	0.9979	0.0001	0.0120	0.0052
8	0.9986	0.9986	0.0001	0.0096	0.0044
9	0.9981	0.9981	0.0001	0.0110	0.0047
10	0.9976	0.9976	0.0002	0.0131	0.0053

**Fig. 18:**
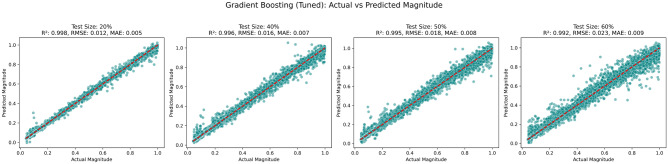
Relationships between the actual and predicted magnitudes.

The scatter plots in Fig. 18 present the relationships between the actual and predicted magnitudes produced by a tuned gradient boosting regression model across four test set proportions (20%, 40%, 50%, and 60%). This analysis demonstrates how simulation resources can be substantially reduced. Notably, in the test case or TC-20 scenario, the gradient boosting model, Which was tested on 80% of the simulation data, achieved the highest predictive accuracy (R$^2$ = 0.998, RMSE = 0.012, MAE = 0.005). Similarly, in TC-40, a model trained on 60% of the data can save 40% of the resources. At the same time, the TC-60 scenario shows that a model trained on 40% of the data can still support a 60% reduction in computational effort.

**Fig. 19:**
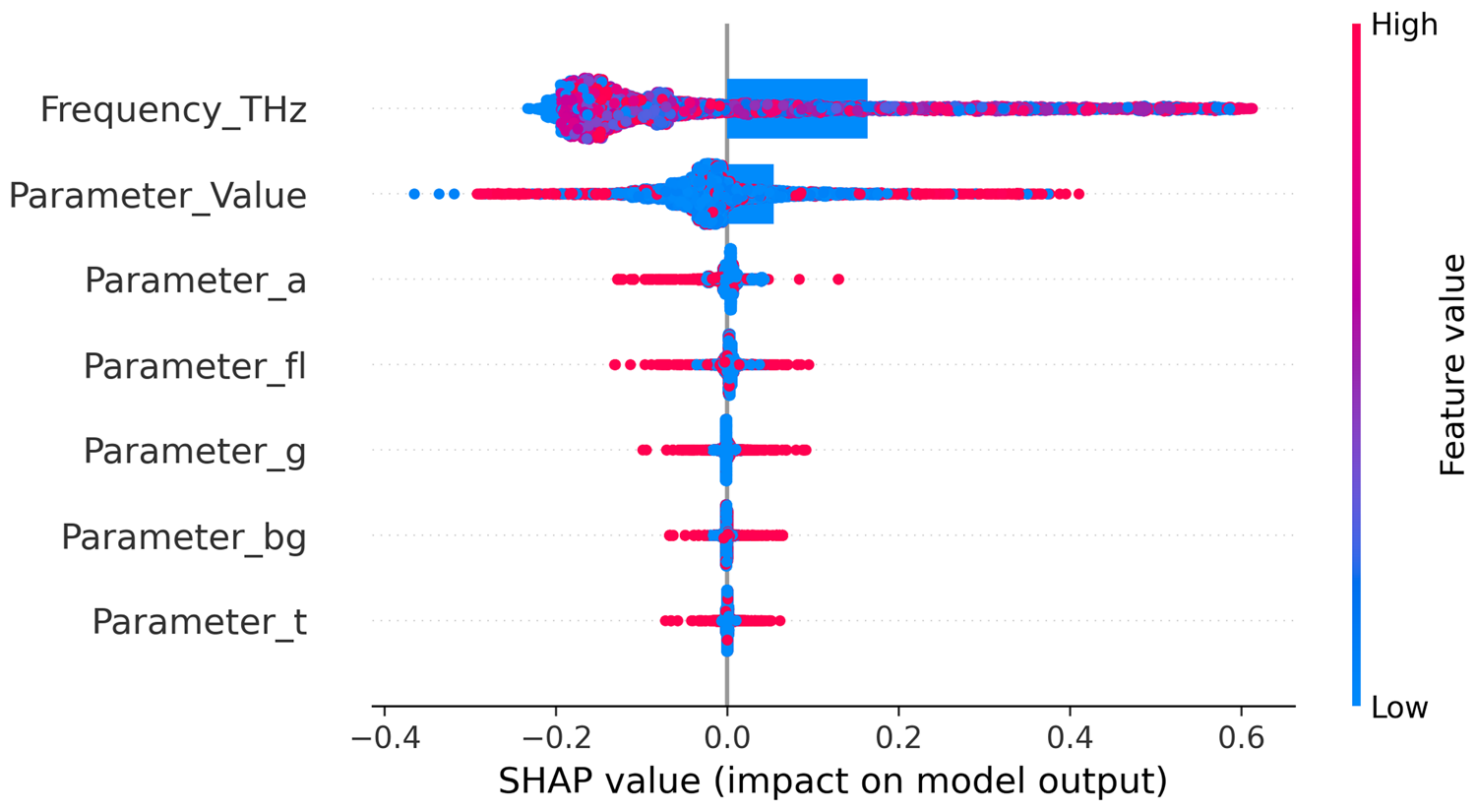
SHAP value impact on output model.

**Fig. 20:**
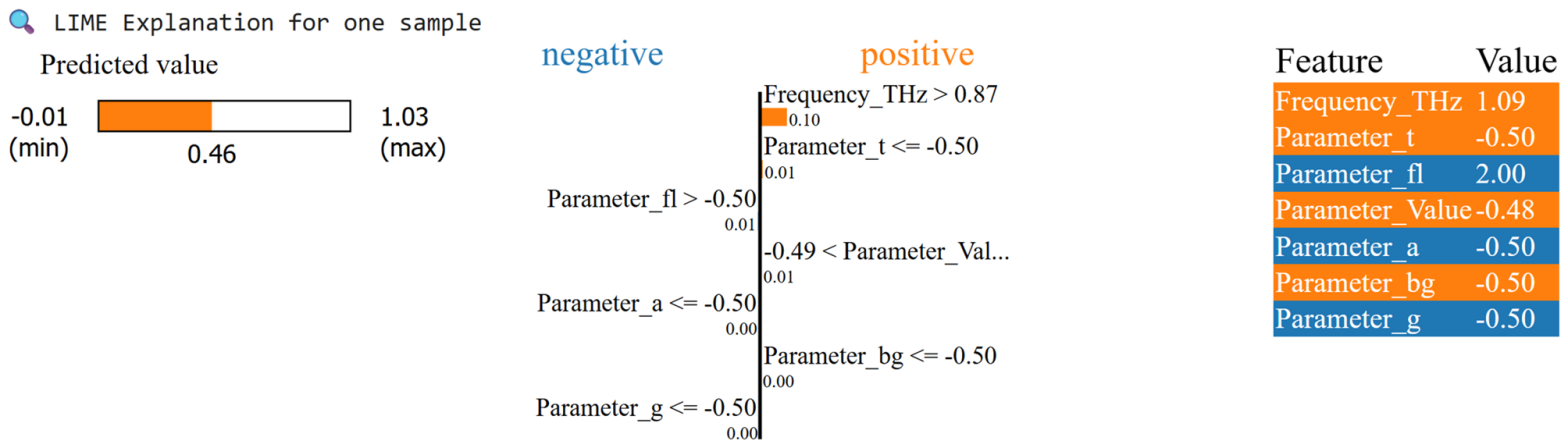
LIME explanation for the GradientBoosting model.

The relative importance of input parameters concerning magnitude for the gradient boosting model is evaluated via SHAP (Fig. 19) and LIME (Fig. 20). The SHAP beeswarm plot displays features ordered by the average absolute SHAP value, with a blue-to-red color indicating low-to-high feature values, and horizontal spread indicating positive or negative impacts on magnitude^69^. In both the SHAP and LIME visualizations, Frequency_THz is the primary factor influencing the prediction magnitude. The SHAP beeswarm plot has the widest spread and the highest average absolute impact, making it the most globally influential feature. Locally, for a particular test instance where Frequency_THz = 1.09, the model output shifts significantly upward from the baseline ( 0.46), which is a much greater effect than that of other features. The following two key contributors in SHAP are Parameter_fl = 2.00 and Parameter_a = –0.50, which provide notable but smaller positive and negative influences. LIME confirms these results: it assigns the most significant weight in its surrogate model to Frequency_THz ($\approx$ +0.63), followed by Parameter_fl ($\approx$ + 0.22) and Parameter_Value ($\approx$ + 0.18), emphasizing Frequency_THz’s dominance in shaping the prediction.

Overall the sensor provide an overall outstanding results for it’s high absorption and sensitivity as well as it’s performance in differentiating several brain tissue conditions. Moreover, the ML models can help to reduce the simulation time upto 60% and helps to optimize the design structure if needed.

Although the study is entirely simulation-driven, the design has been validated using ADS through equivalent circuit modeling, with practical fabrication considerations kept in mind during development. Potential microfabrication methods that could be used to implement this structure in practice are also outlined. To evaluate its performance in a practical measurement setup, a dry phantom that mimics the electromagnetic properties of biological tissues will be employed as a preliminary step to optimize sensor response prior to human testing. Future work will focus on applying the sensor for disease detection, with human sample testing contingent upon ethical approval, and subsequent clinical trials assessing diagnostic performance through metrics such as sensitivity, specificity, and predictive accuracy. The overall results, when compared with previous works, demonstrate the superior performance of the proposed sensor, making it an ideal technology for advancing the detection of neurological disorders.

## Conclusion

This work presents a triple-band metamaterial structure featuring a combination of square and octagonal shapes for preliminary detection of brain-related disorders. The structure is straightforward and compact, measuring $35\times 35 \times 3.8 \upmu m^3$, and operates at frequencies of 4–8 THz. The absorption values are very high, at 99.8%, 99.3%, and 99.8% for frequencies of 4.632 THz, 6 THz, and 7.384 THz, respectively. The sensitivity test results for different refractive indices are excellent, 1.5 THz/RIU, 1.5 THz/RIU, and 1.8 THz/RIU demonstrate the reliability of the sensor for sensing. The Q-factor and FOM values were also evaluated, with Q-factors of 22.21, 29.41, and 33.81, and corresponding FOM values of 7.19, 7.35, and 8.24, respectively, for these frequencies. Furthermore, E-field, H-field, and surface current analyses, as well as an equivalent circuit, are developed via ADS and compared with the simulation results. Several machine learning models, including Gradient Boosting and XGBoost, were applied to predict the design structure. Among them, Gradient Boosting demonstrated high accuracy and showed that up to 60% of the simulation time could be reduced. The sensor was tested using different refractive index (RI) values of brain tissues, and the results showed that all the channels performed well in differentiating various conditions. Given its compact size and outstanding performance, the proposed sensor holds significant potential as a reliable tool for detecting early-stage neurological disorders.

## Supplementary Information

Supplementary Information.

## Data Availability

The data supporting the findings of this study are provided in the Supplementary Information files. Additionally, the dataset used for training and validating the machine learning models is available on GitHub at: https://github.com/arasad28/Machine-Learning-Enhanced-Multi-Band-mtm-Sensor-for-Early-Detection-of-Neurological-Disorder
